# Cortical priming strategies for gait training after stroke: a controlled, stratified trial

**DOI:** 10.1186/s12984-020-00744-9

**Published:** 2020-08-17

**Authors:** Sangeetha Madhavan, Brice T. Cleland, Anjali Sivaramakrishnan, Sally Freels, Hyosok Lim, Fernando D. Testai, Daniel M. Corcos

**Affiliations:** 1 Department of Physical Therapy, Brain Plasticity Lab, University of Illinois at Chicago, 1919 W. Taylor St, Chicago, IL 60612 USA; 2 University of Illinois at Chicago, Epidemiology and Biostatistics, Chicago, IL USA; 3 University of Illinois at Chicago, Department of Neurology and Rehabilitation, Chicago, IL USA; 4 Northwestern University, Physical Therapy & Human Movement Sciences, Chicago, IL USA

**Keywords:** tDCS, TMS, Motor priming, Treadmill training, Locomotion, High intensity

## Abstract

**Background:**

Stroke survivors experience chronic gait impairments, so rehabilitation has focused on restoring ambulatory capacity. High-intensity speed-based treadmill training (HISTT) is one form of walking rehabilitation that can improve walking, but its effectiveness has not been thoroughly investigated. Additionally, cortical priming with transcranial direct current stimulation (tDCS) and movement may enhance HISTT-induced improvements in walking, but there have been no systematic investigations. The objective of this study was to determine if motor priming can augment the effects of HISTT on walking in chronic stroke survivors.

**Methods:**

Eighty-one chronic stroke survivors participated in a controlled trial with stratification into four groups: 1) control–15 min of rest (*n* = 20), 2) tDCS–15 min of stimulation-based priming with transcranial direct current stimulation (*n* = 21), 3) ankle motor tracking (AMT)–15 min of movement-based priming with targeted movements of the ankle and sham tDCS (*n* = 20), and 4) tDCS+AMT–15 min of concurrent tDCS and AMT (n = 20). Participants performed 12 sessions of HISTT (40 min/day, 3 days/week, 4 weeks). Primary outcome measure was walking speed. Secondary outcome measures included corticomotor excitability (CME). Outcomes were measured at pre, post, and 3-month follow-up assessments.

**Results:**

HISTT improved walking speed for all groups, which was partially maintained 3 months after training. No significant difference in walking speed was seen between groups. The tDCS+AMT group demonstrated greater changes in CME than other groups. Individuals who demonstrated up-regulation of CME after tDCS increased walking speed more than down-regulators.

**Conclusions:**

Our results support the effectiveness of HISTT to improve walking; however, motor priming did not lead to additional improvements. Upregulation of CME in the tDCS+AMT group supports a potential role for priming in enhancing neural plasticity. Greater changes in walking were seen in tDCS up-regulators, suggesting that responsiveness to tDCS might play an important role in determining the capacity to respond to priming and HISTT.

**Trial registration:**

ClinicalTrials.gov, NCT03492229. Registered 10 April 2018 – retrospectively registered, https://clinicaltrials.gov/ct2/show/NCT03492229.

## Background

Many stroke survivors experience chronic gait impairments. After rehabilitation, 36% of stroke survivors cannot walk independently [1] and walk ~ 50% slower than age-matched peers [2], which is well below the speed required for safe community ambulation (1.06 m/s) [3]. Walking endurance is also markedly reduced after stroke [4]. Reduced walking speed and endurance are major barriers for community participation [4, 5] and are associated with decreased physical activity [6] and quality of life [7, 8]. Gait rehabilitation has focused on development of interventions to restore ambulatory capacity, and there is a critical need to maximize benefits of current walking training interventions.

Because of its clinical and home accessibility, treadmill training has long been utilized as an effective and feasible method of walking training for post-stroke individuals [9]. Recent studies have explored high-intensity interval training (HIIT) as a way to reduce training time and volume while maximizing intensity [10]. HIIT involves alternating periods of walking at a high intensity and recovery intensity. Speed-based HIIT (HISTT) is one type of HIIT designed to improve an individual’s walking speed by training at the maximum tolerated treadmill belt speed. HISTT leads to greater improvements in overground walking speed than progressive treadmill training in chronic [11, 12] and sub-acute [13] stroke. The effectiveness of HISTT at improving clinical gait and neurophysiological outcomes post-stroke has not been thoroughly investigated.

Cortical priming with neurostimulation or movement is a promising adjuvant therapy to enhance effects of motor rehabilitation [14]. The premise behind neural priming is that the brain retains its capacity to reorganize after stroke, and priming may improve the effect of associated motor training by correcting the imbalance in interhemispheric inhibition observed post-stroke and facilitating long-term potentiation and depression like mechanisms [14]. One clinically translatable type of cortical priming is non-invasive transcranial direct current stimulation (tDCS). After stroke, tDCS has been used to correct interhemispheric imbalance in two primary ways: 1) anodal tDCS, in which the anode is placed over the ipsilesional hemisphere to increase ipsilesional cortical excitability and 2) cathodal tDCS, in which the cathode is placed over the contralesional hemisphere to decrease contralesional cortical excitability. Anodal tDCS has been shown to upregulate corticomotor pathways and improve motor learning and function [14–16]. Although cathodal tDCS has also shown beneficial effects, suppressing the contralesional hemisphere may be maladaptive in individuals with limited neural resources in the ipsilesional hemisphere [16, 17]. These findings support the role of tDCS to potentially enhance the effects of other types of motor training [15]. Movement-based priming, another priming technique, often involves the performance of a repetitive movement, such as wrist flexion and extension, prior to performance of motor training [18]. Like tDCS, movement-based priming may also increase corticomotor excitability (CME) and enhance the effects of motor training [18, 19]. Thus, movement-based priming is also a potential adjuvant to enhance the effects of HISTT, and combining tDCS and movement-based priming may yield greater benefits than either type of priming in isolation.

Despite the potential benefits of tDCS and movement-based priming, there have been no systematic investigations on the effects of motor priming on HISTT-induced improvements in walking. Our lab recently found that a single session of HISTT paired with tDCS and movement priming increases excitability of the ipsilesional hemisphere and decreases excitability of the contralesional hemisphere, supporting the potential efficacy of priming for enhancing HISTT [20]. In this controlled trial with stratification, our objective was to determine if motor priming can augment the effects of HISTT. As it is critical that the optimal priming technique be paired with gait rehabilitation, we compared the effects of three types of priming techniques on 4-weeks of HISTT: tDCS, movement-based priming, and both combined. Based on pilot data, we hypothesized that tDCS and movement-based priming would enhance the effects of HISTT on walking speed with corresponding changes in CME and combining both types of priming would lead to even greater improvement.

## Methods

This study was approved by the institutional review board at the University of Illinois at Chicago (UIC), and all participants provided written informed consent. Trial registration: ClinicalTrials.gov, NCT03492229. Registered 10 April 2018 – retrospectively registered as data collection was initiated prior to the revised NIH clinical trial registration guidelines, https://clinicaltrials.gov/ct2/show/NCT03492229.

Participants had sustained a single, monohemispheric stroke > 6 months prior, were 40–80 years old, had residual gait deficits but could walk without external aid for 5 min, had ≥5° active dorsiflexion in the paretic ankle necessary to perform movement priming, and had a Mini-Mental State Examination (MMSE) score of > 21 to ensure they could follow instructions. Participants were excluded if they had brainstem or cerebellar lesions, a score of ≥2 on the Modified Ashworth Scale, were taking uncontrolled spasticity medications, had major cardiorespiratory or metabolic diseases, or had contraindications to transcranial magnetic stimulation (TMS), including history of seizures, implanted metallic objects, and use of medications that alter cortical excitability.

This was a single-center, multi-arm trial with four parallel groups. Participant group was selected with minimization and an allocation ratio of 1:1:1:1. Minimization required a similar number of participants in each group within age, speed, and paretic Fugl Meyer lower extremity assessment (FMLE) score ranges. Age ranges: ≤50, 51–60, 61–70, and 71–80 years; speed ranges: 0.1–0.4, > 0.4–0.8, and > 0.8 m/s; and FMLE ranges: < 20, 21–25, 26–30, > 30. When multiple groups could achieve minimization, participant group was assigned randomly. A study coordinator performed minimization, enrollment, and allocation. Group assignment was concealed from investigators performing pre-, post-, and 3-month follow-up assessments. Testing and training were performed in the Brain Plasticity Laboratory at UIC.

A power analysis based on pilot data indicated that 20 participants per group were required (α = 0.05, 1-β = 0.80) to detect between group differences in change in walking speed (akin to a group X time interaction effect) of 0.3 m/s. An analysis performed after testing ~ 20 participants per group found no group differences for change in comfortable walking speed (largest conditional power, Z_k_ = 0.08). A futility analysis was performed to determine whether testing 5 additional participants per group would likely yield a significant between group difference [21]. The probability of rejecting a false null hypothesis after the addition of these participants was 0.006% (futility: 0.9994), indicating that there is almost no chance that testing these additional participants would yield a significant between group difference.

### Motor priming

Participants were assigned an intervention group dictating the priming received prior to HISTT: 1) control–15 min of rest, 2) tDCS–15 min of stimulation-based priming with tDCS only, 3) AMT–15 min of movement-based priming with ankle motor tracking (AMT) and sham tDCS, and 4) tDCS+AMT–15 min of concurrent priming with tDCS and AMT.

The AMT and tDCS+AMT groups performed a skilled visuomotor ankle motor control task with the paretic leg, analogous to movement-based priming approaches in the upper limb [18]. Participants were seated comfortably with the paretic leg secured to a custom ankle tracking device and were instructed to perform dorsiflexion and plantarflexion to match ankle position to a moving sinusoid displayed on a screen [22, 23]. The sinusoid had a random frequency (0.2–0.4 Hz) and amplitude (60–80% of each participant’s comfortable range of motion). Two 60-s practice trials and twelve 60-s trials were performed with 60 s of rest after every four trials. In the tDCS+AMT group, tDCS was applied concurrently with ankle tracking. In the AMT group, tDCS electrodes were affixed, but sham stimulation was applied. Ankle position was sampled at 1000 Hz with Spike2 (CED, UK).

Facilitatory tDCS was applied with a constant current stimulator (Chattanooga Iontophoresis, DJO Global, CA, USA). A saline-soaked sponge electrode (anode: 5 × 2.5 cm) was placed over the leg representation of the ipsilesional motor cortex (M1) identified during the pre-test. A carbonized dispersive electrode (cathode: 4.5 × 5.5 cm) was placed over the contralesional supraorbital region. After a 30-s ramp-up, 1 mA current was applied for 15 min. We have demonstrated focality and effectiveness of lower limb tDCS with low current intensities and a small active electrode (1 mA with a ~ 8-cm^2^ electrode vs. standard 2 mA with a 35-cm^2^ electrode). The current density (0.08 mA/cm^2^) and total charge (0.072 C/cm^2^) are comparable to other studies and within safety limits [16]. For sham stimulation in the AMT group, the stimulator was turned off after the 30-s ramp-up.

### High-intensity speed-based treadmill training (HISTT)

Before training, participants received ~ 10 min of lower limb stretching. Participants performed 12 sessions (4 weeks, 3 sessions/week) of HISTT [24]. To start each week, maximal walking speed was assessed with the 10-m walk test (10MWT). Treadmill sessions started (warmup) and ended with 5 min of walking at 50% of the weekly maximal walking speed. After warmup, high-intensity intervals were performed. For each interval, treadmill speed was increased over a 2-min period up to the peak speed that was safe and tolerable. Peak speed was held for 10 s. After each interval, participants walked at their warm-up, recovery speed until heart rate (HR) was within 5 bpm of warmup. If HR did not decrease within 4 min, the treadmill was stopped, and participants stood until HR reached the required level. At the end of intervals and recovery periods, Rating of Perceived Exertion (RPE; Borg 10-point scale) was assessed. Total walking time per session was 40 min, less time during which the treadmill was paused. Peak HR, speed, and distance walked were recorded. For safety, participants wore a harness without body-weight support and were allowed to hold onto handrails. Minimal manual assistance with hip and knee flexion at toe off was provided as needed.

Over the 4-week training period, peak treadmill speed was continuously increased. If the participant could safely maintain the peak speed achieved during an interval, treadmill speed was increased for the subsequent interval. At speeds below 3.3 mph (1.48 m/s), each increase in peak speed was 10% of the previous peak speed. To avoid transition to running, increases in peak speed were relatively smaller when increasing above 3.3 mph. If participants displayed foot dragging, needed excessive manual assistance, displayed other signs of instability, or had an excessive increase in HR during an interval, peak speed was decreased by 10% for the subsequent interval.

### Outcomes

Outcomes were assessed at pre-, post-, and 3-month follow-up assessments. Pre- and post-assessments were performed within 2 days of the first/last training session; the 3-month follow-up was performed ~ 90 days after the last training session to investigate retention. A single laboratory member performed all assessments and was blinded to group assignment.

#### Primary outcome: walking speed

Participants performed two trials of the 10MWT walking at a “normal comfortable” speed. Time to complete trials was recorded with a stopwatch, and walking speed was computed from the mean across trials. Walking was performed without assistive devices whenever possible. Participants also performed two 10MWT trials “as fast as safely possible,” to determine a secondary outcome (maximal walking speed).

#### Secondary outcome: corticomotor excitability (CME)

Muscle activity was recorded from the tibialis anterior (TA) of both legs with surface EMG. The skin was shaved and prepped. A reference electrode was placed over C7. EMG data were sampled with an amplifier system (Bagnoli 8, Delsys, MA, USA; frequency: 2000 Hz, gain: 1000, band pass filter: 20–450 Hz) and recorded with Spike2. Participants performed 3 maximal voluntary isometric contractions (MVIC) of the ankle dorsiflexors. Contractions were held for ~ 5 s and performed bilaterally. Visual feedback and verbal encouragement were provided.

TMS was used to assess CME using established procedures [22, 25, 26]. Participants performed isometric ankle dorsiflexion at 10% MVIC with both limbs. Stimulation was applied from a single-pulse stimulator (Magstim 200, Magstim Inc., MN, USA) through a double-cone coil oriented in the posterior-anterior direction. The TMS coil was systematically moved to identify the “hotspot,” the location with the largest, consistent contralateral motor evoked potentials (MEPs). The active motor threshold was determined as the minimum stimulus intensity eliciting MEPs with a peak-to-peak amplitude of ≥0.1 mV in 4 of 8 trials. Recruitment curves were generated by applying stimulation at seven intensities (6 stimuli at each of 80, 90, 100, 110, 120, 130, and 140% of active motor threshold). MEPs were rectified and the area was calculated from MEP onset to offset. The relation between average MEP area and the corresponding stimulation intensity (% active motor threshold) was described with a linear function. CME was characterized as the slope of this function.

As not everyone responds with upregulation to tDCS [27, 28], we subcategorized participants in the tDCS and tDCS+AMT groups into up-regulators and down-regulators. After completion of recruitment curves during the pre-assessment, tDCS was applied for 15 min at rest. CME was re-assessed after tDCS with TMS at 120% AMT, and the percent change in MEP area was calculated.

#### Other secondary outcomes

- Walking endurance, 6-min walk test (6mWT). Participants walked at their comfortable pace for 6 min, and the distance covered was recorded.
- Static balance and fall risk, Berg Balance Scale (BBS). Participants completed 14 static and dynamic balance activities. Maximal score: 56.
- Health status after stroke, Stroke Impact Scale (SIS). Participants completed a 59-item questionnaire. Strength, hand, ADL/IADL, and mobility domain scores were combined as the SIS-16.

#### Exploratory outcomes

- Mobility, Timed Up and Go Test (TUG). Participants rose from a chair, walked 3 m, turned around a cone, walked back, and sat down. Mean time to complete two trials was recorded.
- Dynamic balance, Mini Balance Evaluation Systems Test (miniBESTest). Participants completed 14 dynamic balance activities. Maximal score: 28.
- Motor impairment, FMLE. Participants were scored on 7 movement categories. Maximal score: 34.
- Confidence in performing ambulatory activities without falling, Activities-Specific Balance Confidence Scale (ABC). Participants completed a 16-item questionnaire.

### Statistical analysis

We performed intention-to-treat analyses with linear mixed modeling, which accounts for all data and assumes that data is missing at random. Random intercepts accounted for repeated observations, and a compound symmetry repeated covariance type was used. To predict outcomes, linear mixed models used independent factors of time (pre, post, 3-month: 2 degree of freedom (df) test to determine if the 3 time points are equal) and group (control, tDCS, AMT, tDCS+AMT: 3df test). Primary focus of linear mixed modeling was on group X time interactions (6df test). Significant contrasts are reported. To predict variables from treadmill training sessions (average weekly value) and weekly maximal walking speed, linear mixed models used independent factors of week (1–4: 4df test) and group (3df test). Post-hoc pairwise comparisons were performed with Bonferroni correction. Baseline characteristics were compared between groups with 1-way ANOVA and chi-square analysis. Post-hoc group comparisons were performed with Tukey HSD. Cohen’s d was used for effect sizes. Statistical analyses were performed with SPSS Statistics (IBM, NY, USA), with a *P* value considered statistically significant at 0.05.

## Results

**Fig. 1:**
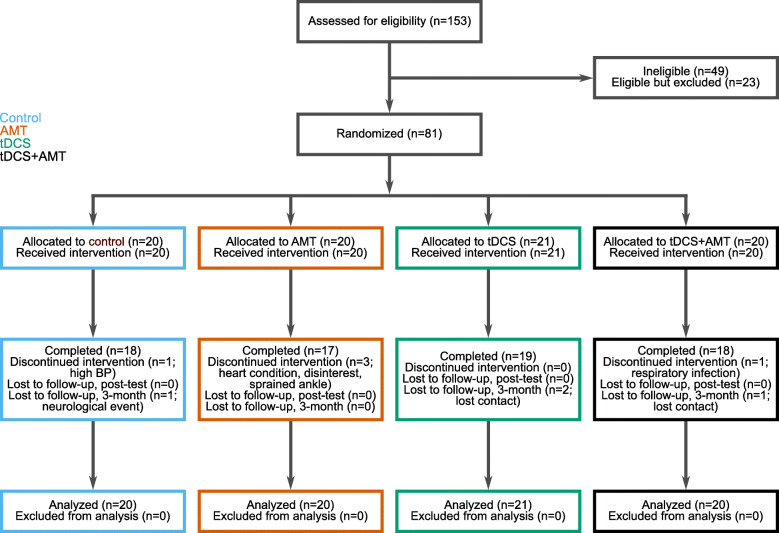
Study flow diagram. Participant flow through the study included eligibility assessment, randomization, allocation, study completion, and inclusion in data analysis. Values in parentheses represent the number of participants that fit each statement. Note the number of analyzed participants matches the number of allocated participants, consistent with an intention-to-treat analysis

Participants were recruited from June 2014 to June 2018, and all assessments were completed by October 2018 (Fig. 1). Groups did not differ at baseline for demographics (Table 1; *p* ≥ 0.48) or outcome measures. Five participants (6%) did not complete training for the following reasons (Fig. 1): blood pressure exceeding 180/100 before HISTT, a heart condition disclosed after enrollment, lost interest, sprained ankle outside of study procedures, and development of an upper respiratory infection. There were no other adverse events during the trial. Compared to participants who completed the study, participants who withdrew were more likely to be women (4 women of 5 withdrawals vs. 22 women of 76 completions; χ^2^ = 5.6, *p* = 0.02) and tended to be younger (51.0 (6.7) vs. 59.2 (9.3) years; t = 1.9; 95% CI: − 0.3, 16.6; *p* = 0.06).

**Table 1:** Demographics

	Control (***n*** = 20)	AMT (n = 20)	tDCS (***n*** = 21)	tDCS + AMT (n = 20)
Age (years)	58 (10)	60 (9)	58 (11)	59 (9)
Sex (female, %)	9 (45%)	5 (25%)	7 (33%)	5 (25%)
Race/Ethnicity (count, %)				
White, Not Hispanic or Latino	6 (30%)	7 (35%)	6 (29%)	8 (40%)
White, Hispanic or Latino	0 (0%)	1 (5%)	3 (14%)	1 (5%)
Asian American	2 (10%)	0 (0%)	2 (10%)	2 (10%)
American Indian	0 (0%)	0 (0%)	0 (0%)	1 (5%)
Black	12 (60%)	12 (60%)	10 (47%)	8 (40%)
More affected limb (right, %)	8 (40%)	7 (35%)	11 (52%)	11 (55%)
Stroke type (ischemic/hemorrhagic)^*^	15/4	11/8	13/8	14/6
Years since stroke	6.1 (4.2)	5.6 (3.6)	4.3 (3.6)	5.9 (5.6)
MMSE	29 (5)	28 (2)	28 (1)	28 (2)

Values assessed at baseline for the control, ankle motor tracking (AMT), transcranial magnetic stimulation (tDCS) and tDCS+AMT groups. Values are mean (SD) or count (%). MMSE: Mini-Mental State Examination. ^*^Information on stroke type was unavailable for two participants.

### Outcomes

#### Walking speed

Data were excluded from one control participant whose change in speed was >4SD above the mean, leaving a sample size of 80 for walking speed analyses.

**Fig. 2:**
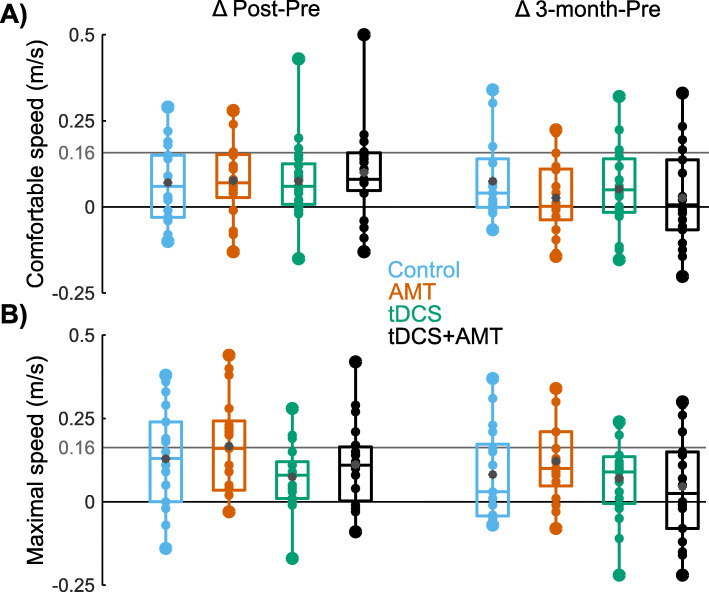
Change in walking speed. Box and whisker plots of change for A) comfortable and B) maximal walking speed from pre- to post-assessment (left column) and from pre- to 3-month assessment (right column). Small dots represent individual data, while large dots represent the minimum and maximum values. Gray dots represent mean values. Boxes range from the 1st to the 3rd quartile, and the middle horizontal lines represent the median values. The gray horizontal line at 0.16 m/s denotes the change in walking speed that meets MCID

- For comfortable 10MWT speed (Table 2, Fig. 2), there was a time effect (F = 19.6, *p* < 0.001) but no group effect (F = 1.5, *p* = 0.21) or group X time interaction (F = 1.0, *p* = 0.41). Comfortable speed was 10% faster at post-assessment (95% CI: 0.05, 0.11; *p* < 0.001; d = 0.37) and 7% faster at 3-month follow-up (95% CI: 0.02, 0.09; *p* < 0.001; d = 0.26) than at pre-assessment. Across the entire sample, comfortable speed increased by 0.08 m/s from pre- to post-assessment. In 17 (21%) participants, comfortable speed increased by ≥0.16 m/s, the minimal clinically important difference (MCID). The proportion of participants reaching MCID from pre- to post-assessment was not different between groups (χ^2^ = 1.8, *p* = 0.61).
- For maximal 10MWT speed (Table 2, Fig. 2), there was a time effect (F = 36.7, *p* < 0.001) but no group effect (F = 2.0, *p* = 0.12) or group X time interaction (F = 1.4, *p* = 0.21). Maximal speed was 12% faster at post-assessment (95% CI: 0.09, 0.16; *p* < 0.001; d = 0.39) and 8% faster at 3-month follow-up (95% CI: 0.05, 0.12; *p* < 0.001; d = 0.26) than at pre-assessment. Between group contrasts showed a greater increase in maximal speed for the AMT than the tDCS+AMT group from pre-assessment to 3-month follow-up (t = 2.0; 95% CI: 0.002, 0.17; *p* = 0.04; d = 0.55). There was also a greater increase in maximal speed for the AMT than the tDCS group from pre- to post-assessment (t = 2.3; 95% CI: 0.01, 0.17; *p* = 0.02; d = 0.75). The proportion of participants (*n* = 25, 31%) reaching MCID from pre- to post-assessment was not different between groups (χ^2^ = 4.6, *p* = 0.20). The tDCS group also had a non-significantly lower proportion of participants meeting MCID than the control group (χ^2^ = 3.5, *p* = 0.06).

**Table 2:** Walking speed and corticomotor excitability

Primary outcome		Group	Pre	Post	3 M	∆ Pre to Post	∆ Pre to 3 M
Comfortable walking speed (m/s)		Control (*n* = 19)	0.77 (0.23)	0.84 (0.22)	0.86 (0.24)	0.07 (0.11)	0.08 (0.12)
		AMT (*n* = 20)	0.74 (0.20)	0.81 (0.21)	0.77 (0.23)	0.08 (0.11)	0.03 (0.11)
		tDCS (n = 21)	0.70 (0.20)	0.77 (0.21)	0.76 (0.26)	0.07 (0.11)	0.06 (0.12)
		tDCS+AMT (*n* = 20)	0.83 (0.21)	0.93 (0.23)	0.86 (0.23)	0.10 (0.14)	0.04 (0.14)
**Secondary outcomes**	**Limb**	**Group**	**Pre**	**Post**	**3 M**	**∆ Pre to Post**	**∆ Pre to 3 M**
Maximal walking speed (m/s)		Control (n = 19)	1.01 (0.33)	1.13 (0.34)	1.10 (0.36)	0.13 (0.15)	0.08 (0.14)
		AMT (*n* = 20)	0.95 (0.25)	1.12 (0.30)	1.07 (0.28)	0.17 (0.15)	0.12 (0.12)
		tDCS (n = 21)	0.88 (0.32)	0.95 (0.33)	0.94 (0.37)	0.08 (0.09)	0.07 (0.12)
		tDCS+AMT (n = 20)	1.10 (0.25)	1.21 (0.30)	1.13 (0.31)	0.11 (0.13)	0.04 (0.16)
Active motor threshold (%MSO)	Paretic	Control (n = 20)	53.1 (10.6)	54.8 (10.0)	52.8 (8.4)	1.7 (3.9)	−0.3 (5.6)
		AMT (n = 20)	56.0 (11.9)	58.4 (11.6)	57.1 (9.9)	2.4 (3.8)	1.1 (7.7)
		tDCS (n = 21)	55.0 (7.6)	55.7 (7.0)	56.9 (7.5)	0.7 (5.9)	1.8 (7.0)
		tDCS+AMT (n = 20)	50.9 (10.9)	52.2 (12.4)	49.9 (12.3)	1.4 (4.8)	−0.9 (5.7)
	Non-Paretic	Control (n = 20)	46.7 (12.2)	45.2 (9.9)	45.1 (9.1)	−1.5 (4.7)	−1.5 (6.4)
		AMT (n = 20)	46.3 (9.3)	45.4 (9.2)	45.6 (10.0)	−0.9 (4.7)	− 0.7 (6.2)
		tDCS (n = 21)	43.2 (8.6)	44.0 (8.2)	44.2 (8.7)	0.8 (4.2)	1.1 (5.7)
		tDCS+AMT (n = 20)	50.9 (10.9)	52.2 (12.4)	49.9 (12.3)	1.4 (4.8)	−0.9 (5.7)
Slope (mV*ms/%threshold)	Paretic	Control (n = 20)	0.07 (0.08)	0.09 (0.08)	0.06 (0.07)	0.02 (0.06)	−0.01 (0.04)
		AMT (n = 20)	0.07 (0.08)	0.04 (0.10)	0.10 (0.10)	−0.02 (0.11)	0.03 (0.11)
		tDCS (n = 21)	0.14 (0.25)	0.10 (0.15)	0.11 (0.12)	−0.04 (0.16)	−0.03 (0.19)
		tDCS+AMT (n = 20)	0.07 (0.10)	0.13 (0.26)	0.11 (0.23)	0.07 (0.23)	0.05 (0.17)
	Non-Paretic	Control (n = 20)	0.11 (0.10)	0.13 (0.09)	0.10 (0.08)	0.02 (0.11)	−0.01 (0.13)
		AMT (n = 20)	0.13 (0.18)	0.09 (0.06)	0.13 (0.15)	−0.04 (0.07)	0.01 (0.16)
		tDCS (n = 21)	0.09 (0.04)	0.11 (0.07)	0.14 (0.07)	0.03 (0.06)	0.05 (0.08)
		tDCS+AMT (n = 20)	0.07 (0.10)	0.13 (0.26)	0.11 (0.23)	0.07 (0.23)	0.05 (0.17)

Comfortable and maximal 10-m walk test (10MWT) speeds, active motor threshold (AMT) and the slope of the motor evoked potential (MEP) amplitude recruitment curve are shown for the pre-, post-, and 3-month (3 M) assessments for the control, ankle motor tracking (AMT), transcranial magnetic stimulation (tDCS) and tDCS+AMT groups. Active motor threshold is presented as the percent of maximal stimulator output (MSO). Slopes are presented as mV*ms relative to the percent of the active motor threshold. Also shown are differences between assessments. Values are mean (SD).

#### Corticomotor excitability (CME)

TMS was not performed on 9 participants because of participant-reported discomfort. Of the remaining 72 participants, MEPs in the paretic limb were present in 35 (49%; counts per group: control – 10, AMT – 8, tDCS – 9, tDCS+AMT – 8). Only participants with MEPs were included in analyses of the paretic limb, and they had a higher baseline FMLE score than individuals without MEPs (24.1 (3.0) vs. 18.6 (3.8); t = 6.8; 95% CI: 3.9, 7.1; *p* < 0.001). All participants had contralateral MEPs in the non-paretic limb. There was no group effect (F ≤ 0.9, *p* ≥ 0.47), time effect (F ≤ 0.7, *p* ≥ 0.48), or group X time interaction (F ≤ 1.4, *p* ≥ 0.21) for active motor threshold or the slope of the MEP amplitude recruitment curve.

- For paretic active motor threshold, there was no group effect (F = 1.4, *p* = 0.27), time effect (F = 2.2, *p* = 0.13), or group X time interaction (F = 2.0, *p* = 0.09). Contrasts showed that from pre-assessment to 3-month follow-up, active motor threshold decreased in the tDCS+AMT group but increased in the AMT group (t = 2.1; 95% CI: 0.3, 10.0; *p* = 0.04; d = 1.2). From post-assessment to 3-month follow-up, active motor threshold increased in the tDCS group but decreased in the tDCS+AMT (t = 2.3; 95% CI: 0.6, 10.1; *p* = 0.03; d = 0.92) and control (t = 2.2; 95% CI: 0.5, 10.0; *p* = 0.03; d = 0.98) group. See Table 2.
- For the slope of the paretic MEP recruitment curve, there was no group effect (F = 1.2, *p* = 0.33), time effect (F = 0.2, *p* = 0.83), or group X time interaction (F = 1.7, *p* = 0.15). Contrasts showed that from pre- to post-assessment, slope increased in the tDCS+AMT group but decreased in the tDCS (t = 2.4; 95% CI: 0.03, 0.42; *p* = 0.02; d = 0.95) and AMT (t = 2.2; 95% CI: 0.02, 0.41; *p* = 0.03; d = 0.86) group. From pre-assessment to 3-month follow-up, slope increased in the tDCS+AMT group but decreased in the tDCS group (t = 2.5; 95% CI: 0.04, 0.42; *p* = 0.02; d = 0.94). See Table 2.

#### Other secondary outcomes (Table 3)

- For the 6mWT, there was a time effect (F = 15.8, *p* < 0.001) but no group effect (F = 1.6, *p* = 0.21) or group X time interaction (F = 1.6, *p* = 0.15). Walking distance was longer at post-assessment (95% CI: 12.7, 32.5; *p* < 0.001; d = 0.26) and 3-month follow-up (95% CI: 5.1, 25.5; *p* = 0.001; d = 0.18) than at pre-assessment. Contrasts showed less improvement in 6mWT distance from pre-assessment to 3-month follow-up for the tDCS than the control (t = 2.6; 95% CI: − 53.3, − 6.8; *p* = 0.01; d = 0.88) and AMT group (t = 2.1; 95% CI: − 48.4, − 1.6; *p* = 0.04; d = 0.70). There was also less improvement in 6mWT distance from pre- to post-assessment for the tDCS than the AMT group (t = − 2.2; 95% CI: − 48.2, − 2.2; *p* = 0.04; d = 0.79).
- For the BBT, there was a time effect (F = 7.7, *p* = 0.001) but no group effect (F = 0.12, *p* = 0.95) or group X time interaction (F = 0.79, *p* = 0.58). Scores were higher at post-assessment (95% CI: 0.33, 2.2; *p* = 0.004; d = 0.27) and 3-month follow-up (95% CI: 0.41, 2.3; *p* = 0.002; d = 0.29) than at pre-assessment.
- For the SIS-16, there was a time effect (F = 5.9, *p* = 0.003) and group effect (F = 4.4, *p* = 0.006). Scores were higher at 3-month follow-up than at pre- (95% CI: − 18.8, − 1.4; *p* = 0.02; d = 0.23) and post-assessment (95% CI: − 19.5, − 2.4; *p* = 0.007; d = 0.25). The tDCS group had lower scores than the control group (95% CI: − 73.5, − 6.3; *p* = 0.01; d = 0.51).

**Table 3:** Other secondary & exploratory outcome measures

Secondary outcomes	Group	Pre	Post	3 M	∆ Pre to Post	∆ Pre to 3 M
6mWT (m)	Control (*n* = 20)	280 (84)	305 (81)	308 (83)	25 (49)	29 (42)
	AMT (n = 20)	274 (84)	308 (91)	297 (90)	34 (37)	22 (37)
	tDCS (n = 21)	260 (91)	268 (89)	257 (97)	9 (26)	−2 (27)
	tDCS+AMT (n = 20)	307 (87)	329 (87)	319 (94)	22 (37)	13 (44)
BBT	Control (n = 20)	47.8 (4.3)	49.9 (4.3)	50.4 (5.0)	2.2 (3.7)	2.7 (4.7)
	AMT (n = 20)	49.7 (5.1)	50.0 (4.4)	50.4 (4.0)	0.4 (3.8)	0.8 (3.5)
	tDCS (n = 21)	48.9 (4.6)	50.0 (5.0)	49.7 (4.8)	1.2 (3.0)	0.8 (3.2)
	tDCS+AMT (n = 20)	49.1 (5.3)	50.4 (4.0)	50.3 (3.7)	1.4 (3.7)	1.4 (3.7)
SIS-16	Control (n = 20)	212 (53)	228 (52)	229 (64)	16 (24)	18 (43)
	AMT (n = 20)	192 (47)	194 (44)	200 (42)	1 (35)	7 (39)
	tDCS (n = 21)	177 (34)	190 (38)	182 (34)	22 (42)	5 (46)
	tDCS+AMT (n = 20)	209 (32)	222 (38)	219 (38)	13 (26)	10 (18)
**Exploratory outcomes**						
TUG (s)	Control (n = 20)	14.1 (4.9)	13.4 (5.0)	13.9 (5.0)	−0.7 (1.9)	−0.3 (1.8)
	AMT (n = 20)	14.9 (3.3)	13.9 (3.3)	14.2 (4.1)	−1.0 (1.7)	−0.7 (2.8)
	tDCS (n = 21)	17.3 (7.0)	16.2 (7.0)	16.8 (7.5)	−1.1 (2.8)	−0.6 (2.3)
	tDCS+AMT (n = 20)	14.6 (5.5)	13.4 (5.1)	15.2 (7.1)	−1.1 (1.9)	0.5 (2.7)
miniBESTest	Control (n = 20)	18.3 (4.7)	20.0 (4.5)	19.0 (4.5)	1.7 (3.7)	0.8 (3.8)
	AMT (n = 20)	18.5 (3.9)	19.7 (4.3)	19.8 (4.2)	1.3 (2.7)	1.4 (2.4)
	tDCS (n = 21)	17.2 (4.7)	18.6 (4.9)	17.7 (4.4)	1.3 (2.7)	0.5 (2.8)
	tDCS+AMT (n = 20)	19.4 (3.9)	20.0 (3.9)	20.5 (4.0)	0.7 (3.1)	1.1 (2.8)
FMLE	Control (n = 20)	21.1 (5.1)	21.6 (5.7)	22.4 (5.5)	0.5 (4.0)	1.3 (3.5)
	AMT (n = 20)	21.4 (3.3)	21.7 (4.7)	22.4 (4.4)	0.2 (3.6)	1.1 (2.7)
	tDCS (n = 21)	20.0 (4.7)	20.2 (3.8)	20.6 (4.1)	0.1 (3.0)	0.7 (3.1)
	tDCS+AMT (n = 20)	22.0 (4.0)	22.3 (3.8)	24.0 (3.9)	0.3 (3.0)	2.1 (4.1)
ABC	Control (n = 20)	73 (16)	74 (15)	76 (18)	1 (13)	2 (17)
	AMT (n = 20)	71 (18)	73 (13)	75 (14)	1 (14)	4 (12)
	tDCS (n = 21)	69 (16)	72 (18)	72 (17)	3 (15)	3 (14)
	tDCS+AMT (n = 20)	76 (17)	79 (11)	76 (15)	4 (10)	0 (12)

Secondary outcome measures were 6-min walk test (6mWT), Berg Balance Test (BBT), and Stroke Impact Scale (SIS). Exploratory outcome measures were Timed Up and Go (TUG), Mini Balance Evaluation Systems Test (miniBESTest), Fugl Meyer Assessment of lower extremity motor function (FMLE), and Activities- Specific Balance Confidence Scale (ABC). Scores are shown for the pre-, post-, and 3-month assessments for the control, ankle motor tracking (AMT), transcranial magnetic stimulation (tDCS) and tDCS+AMT groups. SIS scores are transformed to represent the percentage of the highest possible score. The SIS-16 combines scores from the strength, activities of daily living/instrumental activities of daily living (ADL/IADL), mobility, and hand subscales. Values are mean (SD).

#### Exploratory outcomes (Table 3)

- For TUG, there was a time effect (F = 8.4, *p* < 0.001) but no group effect (F = 1.2, *p* = 0.31) or group X time interaction (F = 1.0, *p* = 0.41). TUG was faster at post- than pre-assessment (95% CI: − 1.6, − 0.4; *p* = 0.001; d = 0.18) and 3-month follow-up (95% CI: − 1.5, − 0.2; *p* = 0.007; d = 0.15).
- For the miniBESTest, there was a time effect (F = 6.9, *p* = 0.001) but no group effect (F = 1.1, *p* = 0.38) or group X time interaction (F = 0.6, *p* = 0.75). Scores were higher at post-assessment (95% CI: 0.40, 2.0; *p* = 0.001; d = 0.28) and 3-month follow-up (95% CI: 0.07, 1.7; *p* = 0.03; d = 0.21) than at pre-assessment.
- For FMLE, there was a time effect (F = 5.2, *p* = 0.007) but no group effect (F = 1.4, *p* = 0.26) or group X time interaction (F = 0.4, *p* = 0.88). Scores were higher at 3-month follow-up than at pre-assessment (95% CI: 0.28, 2.2; *p* = 0.006; d = 0.28).
- The ABC scale did not have significant effects (F ≤ 1.7, *p* ≥ 0.19).

### HISTT

**Fig. 3:**
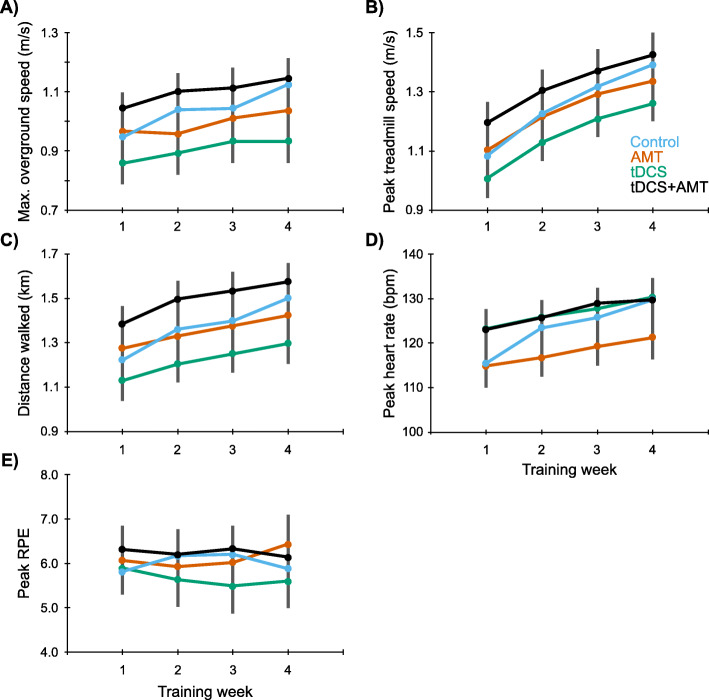
HISTT weekly measures. Weekly values for A) maximal overground walking speed were tested at the start of each training week. Weekly values for B) peak treadmill walking speed, C) distance walked, D) peak heart rate, and E) peak Borg 0–10 Rating of Perceived Exertion (RPE) are the average across the three training sessions within the respective week. Group mean ± SE is shown

Participants completed 5.4 (0.9) intervals per session with 1.5 (1.5) pauses. Across 4 weeks, peak treadmill speed increased from 1.04 (0.30) to 1.37 (0.31) m/s, and walking distance increased from 1.24 (0.40) to 1.36 (0.37) km. Weekly maximal walking speed (F = 19.3, *p* < 0.001), peak treadmill speed (F = 216.3, *p* < 0.001), distance covered (F = 48.1, *p* < 0.001), and peak HR (F = 47.9, *p* < 0.001) increased across sessions (Fig. 3). There were no group effects (F ≤ 1.9, *p* ≥ 0.13) or interactions (F ≤ 1.8, *p* ≥ 0.07). RPE showed no effects (F ≤ 1.1, *p* ≥ 0.33).

### Exploratory analyses: factors impacting response to motor priming

**Fig. 4:**
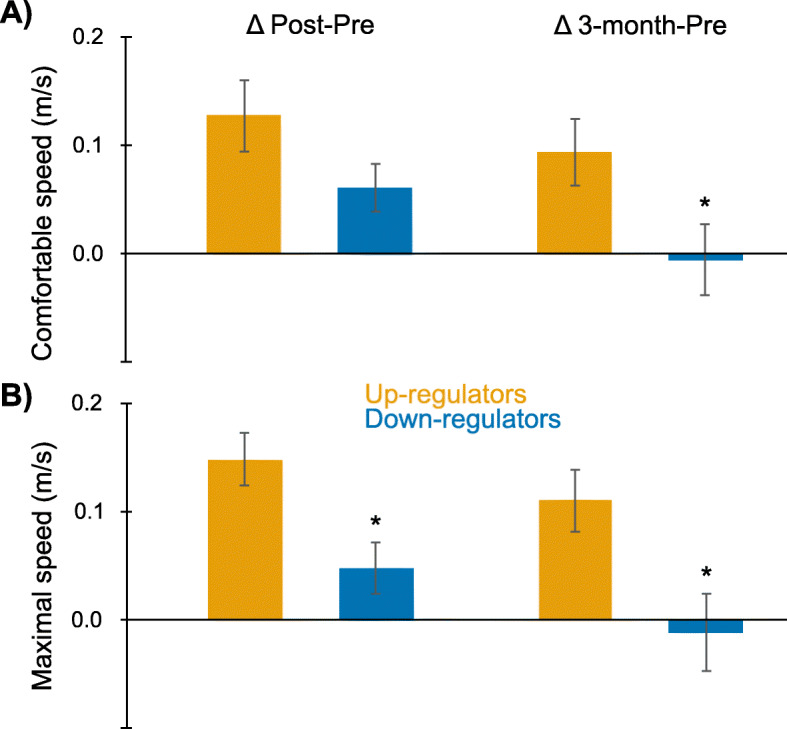
tDCS responsiveness. Box plots of change in A) comfortable and B) maximal walking speed from pre- to post-assessment (left column) and from pre- to 3-month assessment (right column). *p < 0.05 between up-regulators and down-regulators

Individual tDCS responsiveness may have contributed to variability in responses to motor priming (Fig. 4). At pre-assessment in the tDCS and tDCS+AMT groups, 15 min of tDCS led to increased paretic MEP amplitude (> 0% change) in 19 participants (up-regulators) and decreased amplitude (< 0% change) in 17 participants (down-regulators). Up-regulators had a non-statistically significant greater increase in comfortable walking speed from pre- to post-assessment (0.13 vs. 0.06 m/s; 95% CI: − 0.02, 0.15; t = 1.6; *p* = 0.11; d = 0.56) and a significantly greater increase from pre-assessment to 3-month follow-up (0.09 vs. -0.01 m/s; 95% CI: 0.01, 0.19; t = 2.2; *p* = 0.04; d = 0.76) than down-regulators. Up-regulators also had a greater increase in maximal walking speed from pre- to post-assessment (0.15 vs. 0.05 m/s; 95% CI: 0.03, 0.17; t = 3.0; *p* = 0.006; d = 1.01) and from pre-assessment to 3-month follow-up (0.11 vs. -0.01 m/s; 95% CI: 0.03, 0.21; t = 2.7; *p* = 0.01; d = 0.91) than down-regulators.

## Discussion

This is the first study to examine the effects of priming paired with a speed-based, high-intensity gait training paradigm in chronic stroke. Four weeks of HISTT led to improvements in walking speed and endurance, which were partially maintained 3 months after training. Priming with tDCS, AMT, or both did not enhance the effects of HISTT on walking speed, but tDCS+AMT enhanced ipsilesional CME, which was retained at follow-up. Responsiveness to tDCS influenced the effects of priming on HISTT.

HISTT was feasible and effective, improving walking speed and endurance in all groups, with retention 3 months later. These results suggest that HISTT leads to significant and long-term improvements in walking. Consistent with our findings, small-scale studies using aerobic HIIT show improvements in aerobic capacity, walking speed, and walking endurance after stroke [29, 30]. The current study and these previous studies all found ~ 10% improvement in walking speed and endurance following high-intensity training. Improvements in walking speed exceed those from progressive treadmill training [11–13] with less training time, making HISTT a more efficient approach to rehabilitation after stroke. Mobility, balance, and health status also improved with HISTT. Our findings also suggest that longer duration training may be beneficial. After 4 weeks of HISTT, improvements in walking speed did not plateau. From week 3–4, maximal walking speed improved by 0.04 m/s. At this rate, an additional improvement of 0.16 m/s (MCID) could be expected after 8 weeks of HISTT. Studies involving up to 6 months of progressive treadmill training also have not found a plateau in improvements [31, 32]. Future studies should investigate the effects of longer duration HISTT.

After stroke, cortical excitability is often suppressed in the ipsilesional hemisphere and enhanced in the contralesional hemisphere [33], which may be secondary to imbalanced interhemispheric inhibition [34, 35]. We expected tDCS and motor priming to enhance the effects of HISTT because both have been shown to modulate cortical excitability [18, 33, 36–38]. Furthermore, tDCS has been associated with a variety of functional improvements in the lower limb, including enhanced strength, motor control, mobility, and balance [23, 38–41]. However, in the current study, we did not find evidence that combining 4 weeks of HISTT with tDCS, AMT, or both enhances improvements in walking speed or secondary behavioral outcome measures. We may have failed to demonstrate the efficacy of motor priming because HISTT is a strong intervention that masks the effects of priming. Many in the control group had meaningful improvement in comfortable walking speed, indicating that HISTT led to improvements in speed that may have saturated the response potential or masked the effects of motor priming. Additionally, our motor priming interventions were 15 min, while HISTT was 40 min—over 2.5X longer. Motor priming may be more effective when: 1) applied for longer durations, 2) paired with shorter bouts of HISTT, 3) paired with a less intensive intervention, or 4) applied concurrently with or subsequent to HISTT.

Currently, there are 7 published gait studies that used repeated sessions of tDCS paired with walking training for stroke [42–48]. Some of these studies found that tDCS enhanced endurance when paired with robotic gait training [45] and enhanced walking speed when paired with body-weight-supported treadmill training [46]. However, others found that tDCS paired with robotic gait training did not improve walking speed more than gait training alone [42–45, 48]. Similarly, pairing tDCS with standard physical therapy did not enhance improvements in walking for acute or subacute stroke survivors [47, 49, 50]. The study that found a significant effect of tDCS on speed [46] may have done so because the intervention paired with tDCS yielded minimal change in speed (0.04 m/s), allowing identification of priming effects. Although Seo et al. 2017 found a significant effect of tDCS on endurance, they failed to find an effect on speed [45]. Our study expands on these prior studies by pairing tDCS with a higher-intensity intervention (HISTT) and by investigating a larger sample size, and we show no additional benefit of tDCS.

In the current study, we broadly characterized the effects of HISTT and motor priming on walking by quantifying changes in walking speed. Stroke is associated with a number of other changes in walking (e.g. spatiotemporal kinematics, kinetics, and postural stability) which may have been affected by our study interventions. There are no studies evaluating the effect of HISTT on all these walking characteristics, but high-intensity stepping does result in changes in both kinematics and kinetics [51, 52]. Additionally, the application of tDCS over 4 weeks improves postural control as assessed with the Tinetti test [53]. In contrast, other studies suggest that our interventions would not have affected other walking characteristics. A single session of tDCS does not alter spatiotemporal kinematics or kinetics during walking [54–56]. Moreover, other studies have found that combining tDCS with walking training does not alter walking spatiotemporal kinematics [42, 48]. Understanding the impact of HISTT and motor priming on specific walking impairments (e.g. spatiotemporal kinematics, kinetics, and postural stability) is an important area for future research.

The premise of previous work and the current study is that cortical priming will enhance neuroplastic changes in the brain that occur in response to walking training. However, it is well known that spinal central pattern generators are important for walking in humans [57]. Consequently, cortical priming strategies may have a limited benefit, and spinal priming strategies may be more effective. Several studies have investigated this possibility by applying trans-cutaneous spinal direct current stimulation (tsDCS) in conjunction with robotic gait training [48, 58]. These studies found that combining tsDCS with tDCS or trans-cutaneous cerebellar direct current stimulation is more effective than any type of stimulation in isolation for improving walking endurance. These studies suggest that tsDCS may be an important component of future priming strategies.

In our study, the tDCS+AMT group had decreased motor threshold and increased recruitment curve slope in the ipsilesional hemisphere after training, both indicators of increased CME. These effects were retained 3 months after the end of training and are consistent with our finding that a single session of tDCS and HISTT increases CME [20]. Overall, these results suggest that motor priming paired with HISTT leads to long-term enhancement of CME. We may have failed to find other significant differences between groups for TMS measures because MEPs were only available in higher-functioning individuals, presenting a ceiling effect for outcomes derived from TMS. Studies evaluating tDCS modulation of CME have provided mixed results, with some raising questions about the variability and reliability of tDCS [59]. Nonetheless, a recent metanalysis concluded that anodal tDCS significantly improves CME [60]. Interestingly, low currents (≤1 mA) administered for > 10 min (charge of > 0.029 mA/cm^2^) had a greater effect on CME than higher currents. It is still unclear whether these changes in CME benefit functional activities.

We further noticed that 53% of participants receiving tDCS (tDCS and tDCS+AMT groups) had an increase in CME following tDCS. Variability in response to tDCS was expected and is similar to other studies of tDCS responsiveness [27, 28]. These up-regulators had 300% greater improvements in comfortable and maximal walking speed than participants who had a decrease in CME following tDCS (down-regulators). These findings suggest that, in some stroke survivors, applying anodal tDCS to the ipsilesional hemisphere may paradoxically decrease CME, limiting the effectiveness of priming with tDCS. Other investigators have also suggested that applying anodal tDCS to the ipsilesional hemisphere may not aid recovery in all individuals and that neuromodulatory interventions should be individually tailored [61]. Thus, response to tDCS may help identify individuals who would benefit from motor priming. Alternatively, responsiveness to tDCS may also reflect a generalized potential for neuroplasticity and functional improvement. If true, greater improvements in walking speed in tDCS up-regulators may reflect greater responsiveness to HISTT, and not (or in addition to) greater responsiveness to tDCS.

A recent review suggests that tDCS is safe in individuals with stroke, with minimal side effects [62]. Additionally, most studies applying tDCS after stroke have used TMS-based exclusion criteria, which is conservative for tDCS. Thus, it is likely that a large portion of the population could receive this intervention safely. Similarly, several studies support the safety of HIIT in individuals with chronic stroke [63, 64]. Our study is in line with these findings because we found minimal side effects from tDCS or HISTT. Beyond safety, the feasibility of home-based tDCS [65] and HIIT interventions [66] in chronic stroke suggests that these interventions could easily translate to clinical practice.

## Conclusions

HISTT is a feasible and effective gait training paradigm for individuals with chronic stroke. Improvements in walking speed and other behavioral effects elicited with HISTT were not augmented with cortical priming. Combining tDCS with AMT before HISTT led to long-term enhancement of ipsilesional CME compared to tDCS or AMT only, which may have functional benefits. Up-regulation in response to tDCS may be an important predictor of improvements in walking speed following priming plus training. Future studies should investigate whether priming is more efficacious when applied for longer bouts, when paired with shorter bouts of HISTT or a less intensive intervention, when the timing of priming is altered, or when priming is selectively applied to individuals based upon tDCS responsiveness.

## Data Availability

Deidentified data that underlie study results will be shared by the corresponding author upon reasonable request from qualified investigators immediately following publication.

## Abbreviations

10MWT: 10-m walk test
6mWT: 6-min walk test
ABC: Activities-Specific Balance Confidence Scale
AMT: ankle motor tracking
BBS: Berg Balance Scale
CME: corticomotor excitability
EMG: electromyography
FMLE: Fugl Meyer lower extremity assessment
HIIT: high-intensity interval training
HISTT: high-intensity speed-based treadmill training
HR: heart rate
M1: motor cortex
MCID: minimal clinically important difference
MEP: motor evoked potential
miniBESTest: Mini Balance Evaluation Systems Test
MVIC: maximal voluntary isometric contractions
MMSE: Mini-Mental State Examination
RPE: Rating of Perceived Exertion
SIS: Stroke Impact Scale
TA: tibialis anterior
tDCS: transcranial direct current stimulation
tsDCS: transcutaneous spinal direct current stimulation
TMS: transcranial magnetic stimulation
TUG: Timed Up and Go Test
UIC: University of Illinois at Chicago
